# The biogeographic origin of a radiation of trees in Madagascar: implications for the assembly of a tropical forest biome

**DOI:** 10.1186/s12862-015-0483-1

**Published:** 2015-10-05

**Authors:** Sarah Federman, Alex Dornburg, Alexander Downie, Alison F. Richard, Douglas C. Daly, Michael J. Donoghue

**Affiliations:** Department of Ecology and Evolutionary Biology, Yale University, P.O. Box 208106, New Haven, CT 06520 USA; North Carolina Museum of Natural Sciences, Raleigh, NC 27601 USA; Department of Anthropology, Yale University, 51 Hillhouse Avenue, New Haven, CT 06520 USA; New York Botanical Garden, Institute of Systematic Botany, 2900 Southern Boulevard, Bronx, NY 10458 USA

## Abstract

**Background:**

Madagascar’s rain forests are characterized by extreme and uneven patterns of species richness and endemicity, the biogeographic and evolutionary origins of which are poorly understood.

**Methods:**

Here we use a time-calibrated phylogeny of a dominant group of trees in Madagascar’s eastern rain forests, *Canarium*, and related Burseraceae (Canarieae), to test biogeographic hypotheses regarding the origin and radiation of the flora of this unique biome.

**Results:**

Our findings strongly support the monophyly of Malagasy *Canarium*, suggesting that this clade represents a previously undocumented in situ radiation. Contrary to expectations of dispersal from Africa during the Oligocene, concurrent with the formation of Madagascar’s rain forest biome, our analyses support a late Miocene origin for Malagasy *Canarium*, probably by long distance dispersal from Southeast Asia.

**Discussion:**

Our study illustrates the importance of considering long distance dispersal as a viable explanation for clades with pantropical distributions diversifying subsequent to the Oligocene, and it highlights the formation of the Indo-Australian Archipelago and associated fast-moving equatorial surface currents, suggesting an under-appreciated evolutionary link among tropical centers of endemism.

**Conclusions:**

We postulate that the relatively recent establishment and radiation of *Canarium* in Madagascar may have been facilitated by the highly stochastic climates associated with these forest ecosystems.

**Electronic supplementary material:**

The online version of this article (doi:10.1186/s12862-015-0483-1) contains supplementary material, which is available to authorized users.

## Background

While it is widely understood that Madagascar harbors extraordinary levels of endemic and highly threatened biodiversity, rather little is known about the genesis of this diversity [1–3]. What were the source areas for those lineages that radiated in Madagascar and now dominate its distinctive landscapes? How was the biota assembled over time? Answers to such questions are difficult, as Madagascar has been an isolated landmass for the past 88 Ma [3] and, due to continental drift, has experienced major climatic transitions. In the Cretaceous, Madagascar may have had a moist, temperate climate, but during the Paleocene and Eocene, the island passed through the 30° latitude subtropical arid zone and widespread arid environments likely predominated [4, 5]. Subsequently, as Madagascar drifted into the trade-wind belt, moister climates developed on its northern and eastern margins [4]. Under this scenario, the island’s hyper-diverse rain forest biome post-dated dry-spiny forest conditions and likely developed during the Oligocene [4]. This transition to new climatic regimes would have created opportunities both for in situ diversification of existing lineages into novel biomes, and for colonization from tropical biomes elsewhere [6]. While many of Madagascar’s endemic rain forest species have sister lineages within Madagascar, indicating high rates of in situ diversification [1, 3, 7], it is also clear that post-Eocene colonization events have contributed significantly to extant biodiversity [7–9].

During the early development of Madagascar’s rain forests in the Oligocene, predominant marine currents were eastward, favoring colonization from Africa [7]. Yet, there are indications that colonists also arrived from both India and Southeast Asia [1, 3]. Movement by ocean current from Southeast Asia in particular probably became intermittently feasible from the Oligocene (34–23 Ma) [3, 7, 10, 11]. This was due to tectonic shifts in the positions of Indian and Southeast Asian landmasses, and gradual westward reconfiguration of the predominant current [10, 11]. By the early Miocene this reconfiguration was complete, and marine dispersal from Africa to Madagascar would be far less likely [7].

It is widely believed that early arrivers limit the establishment and diversification of subsequent colonizers [12]. This expectation implies that the dominant elements of Madagascar’s tropical forests should be composed mainly of early-arriving African elements. However, Madagascar’s rain forests are unusually stochastic in terms of rainfall, temperature, and exposure to cyclones [5], which may violate theoretical assumptions of relative stability by continually resetting the ecological stage. Could these stochastic forest systems facilitate successful colonization and in situ radiation by late-arriving lineages?

We focus here on *Canarium*, a dominant lineage of trees in Madagascar’s tropical forests, and on related Burseraceae (Canarieae). These plants are widely distributed around the tropics, and *Canarium* species, in particular, often dominate Madagascar’s eastern rain forests, sometimes comprising up to 30 % of the woody biomass at mid-elevation sites [13]. Additionally, *Canarium* fruits provide essential food resources for Strepsirrhine primates including the Aye-Aye (*Daubentonia madagascariensis*) and the critically endangered Black-and-White Ruffed Lemur (*Varecia variegata*). All previous ecological and evolutionary investigations of *Canarium* were predicated on the assumption that there were just two widespread and abundant species [14]. However, a recent taxonomic revision of *Canarium* in Madagascar has fundamentally altered our understanding of these dominant forest trees by positing the existence of 33 species on the island [13]. This new understanding requires a re-investigation of *Canarium*’s ecological role and evolutionary history. Are these species the descendants of one colonist, or more? Did they arrive from Africa during the advent of Malagasy rain forests, as might be expected, or from elsewhere at some other time?

Morphological taxonomic treatments of *Canarium* place the Malagasy taxa within Southeast Asian species complexes [14], which casts doubt on the hypothesis of an Oligocene African origin. Previous phylogenetic studies imply the non-monophyly of *Canarium*, but sampling has been insufficient to address the biogeographic origins of Malagasy *Canarium* [15]. As *Canarium* may not be monophyletic, its evolutionary history must be considered in the context of the Canarieae, a pantropically distributed tropical forest clade of ca. 250 species.

To test competing hypotheses for the biogeographic source(s) of Malagasy *Canarium*, we here infer the most comprehensively sampled phylogeny of Canarieae to date, including 36 % of the clade’s estimated species diversity. We specifically focus on the evolutionary history of Malagasy *Canarium*, providing strong evidence that this is indeed a previously undetected in situ radiation. Using our dated phylogenetic tree as a foundation for biogeographic analyses, we argue that *Canarium* arrived in Madagascar through geologically recent long distance dispersal from Southeast Asia, coincident with the formation of the Indo-Australian Archipelago (IAA). This suggests an under-appreciated evolutionary link among tropical centers of endemism.

## Methods

### Taxon sampling and DNA extraction and sequencing

The Canarieae contains roughly 250 species placed in 11 genera distributed throughout the tropics (Additional file 1: A1), the internal structure of which is currently being revised [16]. We sampled 90 of these species: 13 of *Boswellia*, 50 of *Canarium*, 13 of *Dacryodes*, one of *Garuga*, 11 species of *Santiria*, two of *Trattinnickia*, and the single species of *Triomma* (see Additional file 1: A1 for taxonomic sampling information, A2 for collection and voucher information, and A3 for sequence data obtained from GenBank). This sample encompasses the range of ecological and morphological variation in Canarieae, as well as the major biogeographic regions in which this variation occurs. The majority of the sampled species were collected by the authors (S. Federman, A. Downie, D. Daly), but sampling was also supplemented with previously published sequences available in GenBank (Additional file 1: A2 & A3). Outgroups included representatives of seven other Burseraceae genera and two species of Anacardiaceae used in previous phylogenetic studies of Burseraceae [15, 17, 18].

We sequenced four molecular markers used in previous phylogenetic analyses of Burseraceae [15, 17, 18]: the nuclear ribosomal external transcribed spacer (ETS) and the three chloroplast DNA markers, rbc*L*, rps16, and the trn*L*-*F* intergenic spacer. Plant tissues were ground using the MP Biomedicals FastPrep-24 instrument (Santa Ana, CA), and DNA was extracted using the Qiagen DNeasy plant kit (Valencia, CA) following the manufacturer’s protocol. PCR amplification and sequencing conditions followed Weeks et al. [15]. Sequences were edited using Geneious R7 (http://www.geneious.com), and all new sequences were deposited in GenBank (Additional file 1: A2).

### Sequence alignment and phylogenetic estimation of divergence times

Multiple sequence alignment for each locus was carried out using MUSCLE [19] in Geneious R7 (http://www.geneious.com; Kearse et al. 2012) with each alignment refined by eye. We used PartitionFinder [20] to simultaneously infer both the best-fitting nucleotide substitution models and partitioning scheme. The candidate pool of potential partitions ranged from a single partition per locus to partitions that divided the protein coding loci by codon position.

Bayesian phylogenetic analyses were performed using an uncorrelated lognormal relaxed clock in BEAST v1.7.5 [21] with two independent analyses, each run for 80 million generations (sampled every 8000 generations). Substitution and clock models were unlinked among partitions and a birth-death speciation process on branching times was specified as the tree prior for each analysis [22]. The alignment was partitioned based on the Bayesian Information Criterion (BIC) results inferred in PartitionFinder using the *greedy* algorithm [20]. The best-fit model contained one partition for ETS, one for the chloroplast coding region (rbc*L*), and one for the two non-coding chloroplast regions (rps16 and trn*L*-*F*). A GTR + I+ Γ model of sequence evolution was used for all three partitions [23]. Convergence between runs and adequacy of the burn-in period were both assessed using Tracer v1.5 [21]. Adequate sampling of the posterior distribution was diagnosed by quantification of effective sample size (ESS) values in TRACER, with ESS values above 200 indicating effective sampling [24]. We used Tree Annotator [21] to summarize the posterior probability distribution of trees using a maximum clade credibility tree (MCCT) with median branch lengths.

In order to time-calibrate the phylogeny, we used three fossil-based prior age calibrations and a secondary calibration based on previous divergence-time estimates [18]. These calibrations were explained in detail by Fine et al. [18], and their phylogenetic placements were determined based on morphological assessments of the fossils relative to living members of the Burseraceae by P. Fine and D. Daly [18]. Briefly, the youngest fossil-based calibration was based on endocarps attributed to *Canarium* from Czechoslovakian sediments with an estimated age of 23–29 Ma [25]. Because *Canarium* emerged as non-monophyletic, the *Canarium* fossil was placed with a lognormal probability prior at the least inclusive node containing all of the *Canarium* species sampled (node A), following Fine et al. [18]. The fossil *Protocommiphora europea* from the Bognor and Sheppey sediments of the London Clay, with an estimated age of 48.6 Ma, can be assigned to either *Commiphora* or *Bursera* subgenus *Elaphrium* [26] and was placed with a lognormal probability prior at the most recent common ancestor (MRCA) of *Commiphora* and *Bursera* (node B). The fossil *Bursericarpum aldwickense*, also from the Bognor and Sheppey sediments of the London Clay [26, 27], was assigned with a lognormal probability prior to the MRCA of Protieae (node C). Following Fine et al. [18] and De Nova et al. [17], the age of the MRCA of all Burseraceae (Node D) was constrained using a secondary calibration to place a normally distributed prior age with a mean of 64.92 Ma and a standard deviation of 2.35.

We additionally tested how robust results of the dating analyses were to uncertainty in the phylogenetic placement of the *Canarium* fossils. In particular, we were concerned that fossil placements too deep in the phylogeny might provide false support for generally younger divergence times within the Canarieae [28]. First, we ran a set of analyses incorporating all calibrations except for the *Canarium* fossil as described by Fine et al. [29]. Second, since the signature of historical distributions is often eroded in extant taxa [30–33], we ran a further analysis in which we estimated divergence times using the *Canarium* endocarp fossil as a tip, keeping all other fossil calibrations the same. Since we had no additional morphological data to place this fossil more precisely, we allowed it to vary in position along the stem leading to the crown group at node A.

To account for the possibility of strong support for uncertain nodes in the Bayesian analyses [34, 35], we ran maximum likelihood phylogenetic analyses on the concatenated dataset using 1000 rapid bootstraps in RAxML [36] using identical partitions as the Bayesian analysis and compared the maximum likelihood bootstraps to the Bayesian posterior probabilities as in [37].

### Estimates of colonization pathways

**Fig. 1:**
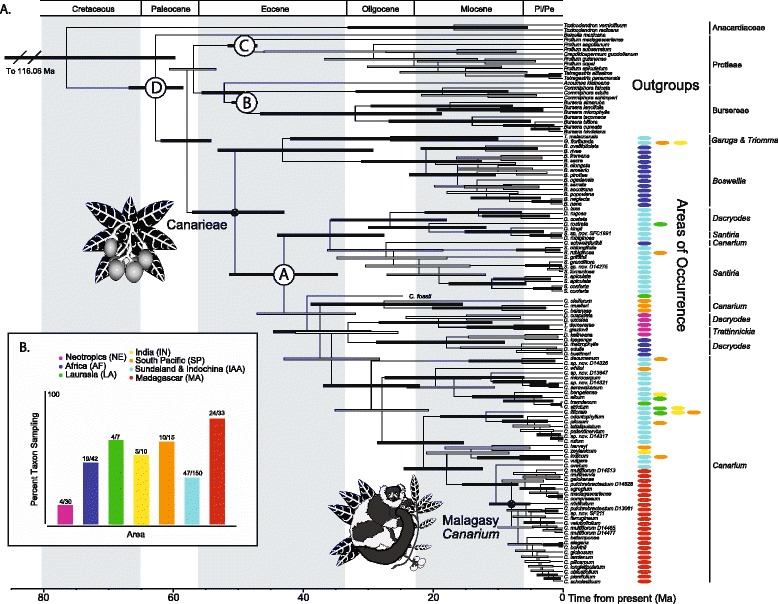
Molecular phylogeny of the Canarieae. **a** Maximum clade credibility tree summarizing the results of Bayesian dating analyses (fossil calibration nodes marked with letters **a**–**d**) with a normal probability prior on node **d** and lognormal probability priors on nodes **a**–**c**. Circled stars mark the crown of the Canarieae and the Malagasy *Canarium* respectively. Taxonomic sections of the Canarieae are noted on the right and colored ovals represent current geographic ranges as defined in (**b**). Bars represent both highest posterior density intervals of the dating analysis as well as Bayesian posterior probabilities (BPP), where black bars represent a BPP of .95 and higher while brown bars represent a BPP between .94 and .70, uncolored bars have a BPP of .69 and below. **b** Table of estimated percent taxon sampling by geographic area

We assigned each species sampled in the phylogeny to one or more of the following seven biogeographic areas: Neotropics (NE); Africa (AF); Sundaland and Indochina (IAA); India (IN); Laurasia (LA); Madagascar (MA); and South Pacific (including New Caledonia, Australia, and Papua New Guinea [SP]) (Fig. 1). These biogeographic areas were delimited on the basis of tropical Asian and African paleogeography [1, 3, 38], and on the distributions of extant species. We used the R package Biogeobears [39] to test the fit of two biogeographic models to our data: (1) the maximum likelihood dispersal extinction cladogenesis model (DEC) [40, 41], and (2) a likelihood version of BayArea [39, 42]. For both of these models, we additionally compared the fit with a founder event parameter, J, which describes a speciation event common to island systems where a “jump dispersal” event quickly results in an evolutionarily independent lineage [39]. Model comparisons were evaluated using AIC scores calculated from each model’s log likelihood (LL). We carried out all analyses on both the fossil-node and fossil-tip calibrated MCCT trees with the outgroups pruned from the MCCT trees prior to analysis.

We used two approaches to model dispersal based on the paleogeographic history of tropical climates from the Eocene onwards. First, we incorporated likely terrestrial and short distance (SD) marine pathways of dispersal through geologic time (hereafter, SD + terrestrial model) that roughly follows previously established models of Madagascar-centric historical biogeography as detailed by Yoder and Nowak [1] and Buerki et al. [3]. We compared this model to one that also takes into account paleoclimatic and paleogeographic information to incorporate possible avenues for long distance marine dispersal (LDD) (hereafter, the LDD + terrestrial model). An advantage to likelihood-based models of biogeography is the ability to partition the temporal history of the clade into time periods with constraints reflecting the climatic and geographic conditions during that time [40, 41]. We divided our models into three time slices-(1) 56–33.9 Ma; (2) 33.9–16 Ma; and (3) 16 Ma-present-and conditioned dispersal rates based on information detailed in the Additional file 1: A4. To account for uncertainty in topology and branch lengths, we chose our best-fitting DEC model, and conducted a statistical DEC model (S-DEC) with the RASP platform [40, 41, 43] using 1000 trees randomly sampled from the posterior distribution of our phylogeny after the burn-in.

### Modeling marine currents

The Oligocene, beginning 34 Ma, corresponds with the earliest evidence of the accumulation of shallow coral diversity in the IAA, reflecting the northward drift of the Australian plate through the Miocene [11, 44, 45]. During this time, currents of the Indian Ocean began to take on their current speed and directionality [7, 10]. To infer the possible role of ocean currents in Canarieae dispersal from the Oligocene onwards, we compiled maps of cumulative mean annual current direction and velocity for El Niño, La Niña, and neutral years using NOAA’s Ocean Surface Current Analyses Real Time (OSCAR) data from 1992 to the present (http://www.oscar.noaa.gov/index.html), and the Marine Geospatial Ecology Toolbox [46] in ArcGIS v10.1. All three cumulative mean annual current maps displayed the same pattern, so we have only included a map of the El Niño years for illustrative purposes (Fig. 3).

## Results

### Phylogenetic analyses and divergence times

Among the three fossil calibration methodologies, divergence times estimated using a fossil-tip taxon were largely congruent with results based on fossil node calibrations both with and without the Canarieae fossil (Fig. 1, Additional file 1: A5 (A) & (B)). While the 95 % highest posterior density intervals (HPD) were largely overlapping between the three phylogenies, there was a shift of the upper bound by ca. 10 Ma, where the fossil-tip calibrated tree inferred older divergence times (Fig. 1, Additional file 1: A5 (A) & (B)). Regardless of the approach (fossil-tip versus fossil node calibrations), our divergence times were congruent with other studies in Burseraceae [17, 18]. Given the similarity between the analyses, we focused our discussion on fossil-tip calibrations, as they accommodated the phylogenetic uncertainty surrounding the placement of the *Canarium* fossil within a non-monophyletic group by allowing it to vary in position along the stem leading to the crown of the wet forest Canarieae (node A).

We found strong evidence for the monophyly of Canarieae with a Bayesian posterior probability (BPP) = 1.0 (Fig. 1). Fossil-tip calibrations estimated a mean crown age for Canarieae at 45.6 Ma with a 95 % HPD of 36.9–54.5. We inferred that sister to the Canarieae were the moist forest Protieae and the predominantly dry forest Bursereae (BPP = 1.0; 95 % HPD = 57.63–65.7). Within Canarieae, the monophyly of the dry forest African *Boswellia* was strongly supported (BPP = 1.0), while *Dacryodes*, *Santiria*, and *Canarium* were inferred as non-monophyletic (Fig. 1), though it is important to note that the Neotropical *Dacryodes* formed a strongly supported clade (BPP = 1.0) with the Neotropical *Trattinnickia* (Fig. 1), with their most recent common ancestor at 20.4 Ma (95 % HPD = 11.6–30.1). Sister to this Neotropical clade was a strongly supported (BPP = 0.99) clade of African *Dacryodes* that diverged ca. 19.0 Ma (95 % HPD = 9.6–30.5). Within *Canarium*, the sole African species (*C. schweinfurthii*) was strongly supported (BPP = 1.0) as being nested within a late Eocene clade of Southeast Asian *Dacryodes* and *Santiria* (mean = 38.11, 95 % HPD = 29.3, 46.7) (Fig. 1). *Canarium schweinfurthii* subtended a clade of Southeast Asian *Santiria*, which, however, was not strongly supported, with a BPP of 0.15 (95 % HPD = 18.9–37.0; Fig. 1).

There was strong support for monophyly of the Malagasy *Canarium* with a crown age of 8.41 Ma (95 % HPD 5.4–11.9). However, relationships within the Malagasy *Canarium* clade were mostly poorly resolved, and species with multiple accessions in the tree were not always monophyletic, possibly due to lineage sorting or hybridization (confident resolution of these relationships will require additional data). This clade was strongly supported as sister to the Southeast Asian *C. ovatum* (BPP = 0.99), with an estimated divergence of 10.9 Ma (95 % HPD = 7.1–16.1) (Fig. 1). All strongly supported clades were also supported in maximum likelihood-based inferences using RaXML (Additional file 1: A6).

### Colonization pathways

Biogeographic model comparisons using the fossil-tip dated phylogeny consistently favored ancestral area estimations based on the LDD + terrestrial model (Table 1). AIC score comparisons of different biogeographic models favored DEC over BayArea, and, for the LDD + terrestrial models, the addition of a founder effect (j). However, inferences of ancestral areas were largely congruent across all analyses (Table 1; Additional file 1: A7 & A8). Inferences of ancestral area conducted over a posterior distribution of 1000 trees from the fossil-tip dated phylogeny using S-DEC were congruent with the best-fit LDD + terrestrial DEC model (Additional file 1: A7), indicating that phylogenetic uncertainty does not effect our inference of the colonization of Madagascar. Here we focus the discussion on those inferences estimated with the best-fit model (DEC + j) for the LDD + terrestrial model; however, the other models tested can be found in Additional file 1: A7 and A8.

**Table 1:** Biogeographic model fits

Model^*^	LnL^a^	P^b^	d^c^	e^d^	j^e^	AIC^f^	dAIC^g^	W^h^
SD + j (BAY)	−136.984	3.000	0.004	0.028	0.003	279.968	52.963	2.0E-12
SD (BAY)	−137.126	2.000	0.004	0.029	0.000	278.253	51.248	4.6E-12
LDD (BAY)	−134.199	2.000	0.003	0.022	0.000	272.397	45.392	8.6E-11
LDD + j (BAY)	−130.529	3.000	0.002	0.016	0.008	267.057	40.052	1.2E-09
SD + j (DEC)	−116.717	3.000	0.007	0.003	0.000	239.434	12.429	0.002
SD (DEC)	−116.716	2.000	0.007	0.003	0.000	237.431	10.426	0.001
**LDD (DEC)**	**−112.009**	**2.000**	**0.004**	**0.001**	**0.000**	**228.018**	**1.012**	**0.370**
**LDD + j (DEC)**	**−** **110.503**	**3.000**	**0.003**	**0.000**	**0.007**	**227.005**	**0.000**	**0.621**

*Results of colonization pathway estimations for SD and LDD models with inferences using DEC versus DEC + j, and BayArea versus BayArea + j. Bolded lines correspond to best fitting models, each of which represent LDD events estimated under DEC and DEC + j, that collectively account for 0.99 of the Akaike weights. SD refers to models incorporating terrestrial and short distance marine pathways of dispersal, while LDD also incorporates long distance marine dispersal pathways; the addition of a + j, indicates that colonization pathways were inferred with the founder event parameter. BAY refers to models estimated using a likelihood version of BayArea while DEC refers to models estimated using a dispersal extinction cladogenesis model

^a^ Log likelihood

^b^ Number of parameters in the model

^c^ Estimated dispersal rate

^d^ Estimated extinction rate

^e^ Estimated founder event speciation rate

^f^ AIC score

^g^ Difference in AIC between the best fit and other candidate models

^h^ AIC weight

**Fig. 2:**
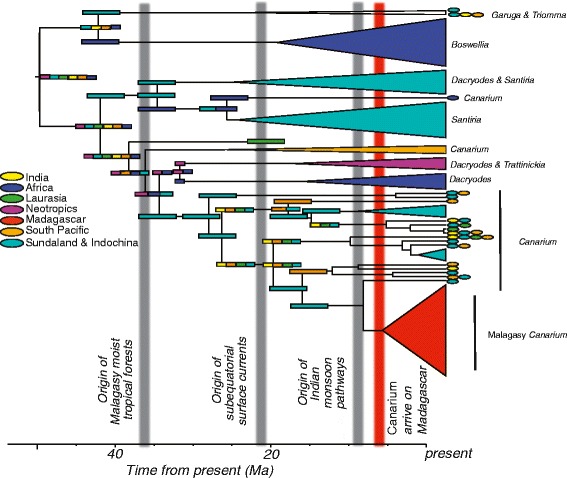
Estimates of geographic range evolution in the Canarieae. Schematic molecular phylogeny of the Canarieae showing estimates of geographic range evolution in seven biogeographic areas: Neotropics; Africa; Sundaland and Indochina; India; Laurasia; Madagascar; South Pacific. Clades represented in a single geographic region are collapsed, while those contemporary taxa occurring in multiple ranges are assigned colored ovals at the tips. Colored bars at interior nodes of the phylogeny correspond to ancestral area reconstructions. Colors corresponding to biogeographic regions are illustrated in the figure legend. Grey bars in the time-scale represent the advent of major geologic and climatic events corresponding to the biogeographic history of the Canarieae, and the red bar corresponds to the crown age of Malagasy *Canarium*

The most likely estimates of the ancestral area for Canarieae suggest that it was fairly cosmopolitan during the Eocene, a time when climates worldwide were generally warmer and drier (Fig. 2). Dispersal via terrestrial habitat tracking and LDD were estimated to be the prevailing forces structuring distribution as opposed to vicariance (Fig. 2). The origin of the African *C. schweinfurthii* was inferred as LDD from Southeast Asia to mainland Africa (Fig. 1). In contrast, range fragmentation of a formerly widespread tropical clade during the late Eocene-early Oligocene likely underlies the origin of both *Dacryodes* and *Boswelia* in Africa. Of comparable age, the Southern Pacific *Canarium* clade was also inferred as having experienced range fragmentation, though within that clade the more recently diverged New Caledonian endemic *C. balansae* was inferred to have achieved its current range via LDD from Australia (Fig. 1).

**Fig. 3:**
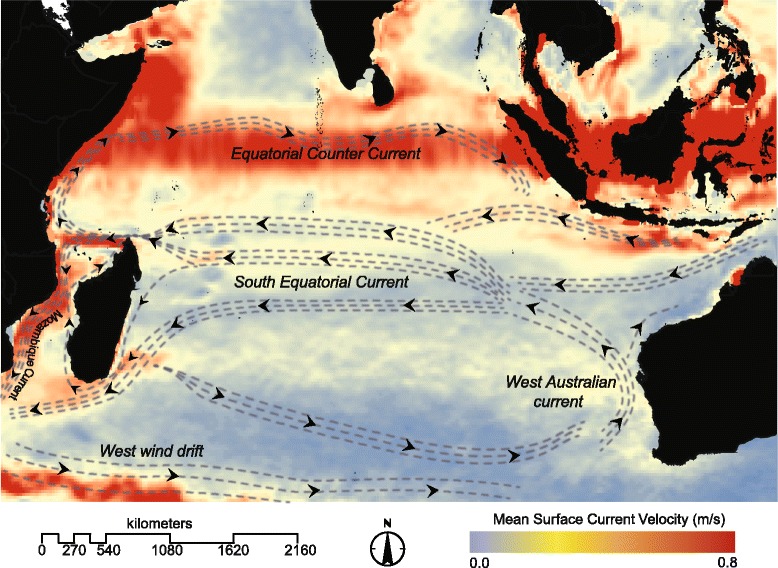
Cumulative mean annual current velocity and direction. Mean annual current velocity of El Niño years from 1992 to the present compiled from NOAA’s Ocean Surface Current Analyses Real Time data. Arrows indicate current direction, while colors indicate current velocity with blue being the slowest and red being the fastest

In the early Oligocene, Canarieae were especially diverse in Southeast Asia, corresponding with the hypothesis of habitat tracking with the gradual cooling of the boreotropics [47]. During the Oligocene and Miocene, there are several instances of possible LDD events with movements from Southeast Asia and the South Pacific westward (Fig. 1). The timing of these movements is broadly concurrent with the formation of the modern IAA (which contains Canarieae’s center of diversity), marine surface currents, and wind patterns [7, 10, 11, 44, 45]. These LDD events include dispersal from Southeast Asia or Australia to New Caledonia, the Tonga Islands, and India for *C. whitei*, *C. harveyi*, and *C. zeylanicum*, respectively (Fig. 1). Importantly, it appears that *Canarium* arrived in Madagascar from Southeast Asia rather than from Africa (consistent with morphological evidence [14]), and that this probably occurred via westward LDD, consistent with estimates of marine dispersal velocity and current pathways between the South Pacific and Madagascar as well as mainland Africa (Fig. 3).

## Discussion

### Pantropical distributions through time

Madagascar and the Southeast Asian Indo-Australian Archipelago (IAA) are two centers of endemism common to many pantropically distributed lineages, Canarieae included [48]. Explanations for these patterns must take into account that these areas are of different ages. For instance, Madagascar has been an isolated landmass since the Cretaceous, while the IAA originated some 100 Ma later [1, 9, 49]. Additionally, as evidenced by *Canarium* in Madagascar (Fig. 1), the ages of many lineages represented in these areas do not correspond with the ages of the areas themselves [1, 3, 49, 50].

Historically, the existence of pantropical lineages was often attributed to Gondwanan vicariance [51, 52]. Under this scenario, lineages represented in multiple centers of endemism must have ancient divergences, and the areas have served as “museums” of biodiversity [53, 54]. However, many clades have proven far too young to owe their current distributions to Gondwanan fragmentation [52, 55–57]. More recently, habitat tracking from the boreotropics to the Southern Hemisphere during Oligocene cooling has become a popular alternative to vicariance biogeography [58–60]. Under this scenario, tropical centers of endemism such as Madagascar and the IAA would represent areas of lineage accumulation with the global contraction of tropical climates. While the tracking of tropical habitats may be a viable explanation for pantropical lineages diverging in the Eocene and early Oligocene, it is less likely for lineages that diverged in the middle Oligocene and Miocene, as these probably post-date the fragmentation and cooling of the boreotropics [47]. For these more recently diverging lineages, LDD may be the more likely explanation [50, 52, 61].

Our results are consistent with the possibility that early divergences in the Canarieae were caused by range fragmentation and tropical habitat tracking in a once cosmopolitan clade. This might apply to the Eocene arrivals in Southeast Asia, to *Boswellia* in Africa, and to the Neotropical *Trattinnickia* and Neotropical and African *Dacryodes* (Fig. 2). These inferences are in line with previous biogeographic studies of Burseraceae [29, 62]. By the late Eocene and early Oligocene, Canarieae had diversified in Southeast Asia, coinciding with rise of the roughly 20,000 landmasses of the IAA and the formation of modern tropical cyclone pathways and fast westward-moving equatorial surface currents (Figs. 2 and 3) that created opportunities for marine LDD [10, 47, 49]. These results are obtained even in the absence of the Canarieae fossil in the dating analysis (Additional file 1: A5B). Indeed, we see evidence in the Oligocene and Miocene that Canarieae moved from the IAA and South Pacific to New Caledonia, India, mainland Africa, and Madagascar (Fig. 2). Interestingly, recent investigations into the origin of Malagasy vertebrates suggest that no colonization events from Asia to Madagascar occurred after the Eocene [63], which highlights the importance of clade-by-clade approaches to understanding the assembly and maintenance of Madagascar’s diverse biota. We suggest that more attention should be paid to the possibility that, in addition to the Canarieae, the formation of the IAA, with its associated fast-moving equatorial currents (Fig. 3) and heavily used migratory flyways [64], could have created a source area for generating the diversity and contemporary distributions of other recently diverged lineages distributed around the wet tropics today [52, 65].

### *The movement of* Canarium *to Madagascar*

Our results strongly suggest that *Canarium* is too young for its existence in Madagascar to be explained by vicariance (Fig. 1); therefore movement to the island most likely occurred via dispersal. There are three plausible dispersal scenarios: (1) LDD via bird dispersal from Southeast Asia; (2) LDD via marine currents from Southeast Asia; and (3) short-distance dispersal, either via bird or water, across the Mozambique Channel, and extinction of African relatives.

As birds are the primary dispersers of *Canarium* in Southeast Asia [16], it is reasonable to suppose that they were responsible for moving *Canarium* to Madagascar. However, we believe that this scenario is less likely than LDD via marine currents. All *Canarium* species, and the majority of the Canarieae (with the exceptions of *Boswellia* and *Garuga*), are dioecious (with separate pollen- and seed-producing plants) [16], and the clade most closely related to Malagasy *Canarium* has relatively large fruits and seeds [14]. In the IAA and South Pacific, *Canarium* fruits are dispersed primarily by large-bodied, non-migratory, non-marine birds such as hornbills and fruit pigeons [16]. For the successful establishment of a dioecious plant, multiple birds, carrying seeds that would give rise to both male and female plants, would need to have made the long trip. Furthermore, although both Madagascar and Southeast Asia fall within important migratory flyways (the east African/west Asian and Asian flyways), these pathways run South and North, overlapping only in Siberia, with no direct pathway between Madagascar and Southeast Asia [64].

Under the marine dispersal scenario, a single fruiting branch (possibly on a natural floating island or tree raft) would be sufficient for male and female plants to become established in close proximity. Westward-moving monsoon pathways originated prior to the inferred movement of *Canarium* to Madagascar [5], as did contemporary warm westward moving currents [10, 11]. Oceanographic investigations show that the Indian Ocean south equatorial current moves at a relatively rapid rate with a phase speed ranging between 15 and 25 cm s^−1^ (Fig. 3). Even in the absence of monsoons, based on current speed alone, dispersal from Sumatra to Madagascar (~6200 Km) would be possible in under 30 days (Fig. 3) [66]. While bird LDD cannot be completely dismissed, we interpret the available evidence as more strongly supporting marine dispersal from the IAA for Malagasy *Canarium*.

An African origin of Malagasy *Canarium* would be feasible only under a scenario in which *Canarium* experienced middle to late Miocene extinctions in mainland Africa, a geologic era when tropical forests contracted with the expansion of the C4 grasslands [67] and tropical forest biodiversity was presumably lost. It is conceivable that a once species-rich clade of African *Canarium* dispersed to Madagascar, but that subsequent extinctions left only a single African species, *C. schweinfurthii* [67, 68]. We note, however, that marine currents during the Miocene were largely unsuitable for marine or animal-mediated dispersal from Africa to Madagascar [7]. In any case, in the absence of fossils such speculations on an African origin are difficult to evaluate, and we favor dispersal from the IAA as the best interpretation of present molecular phylogenetic and morphological evidence, which unequivocally links Malagasy *Canarium* directly with Southeast Asian taxa.

### Development of Madagascar’s rain forests

Our results indicate that *Canarium*, today a dominant element in the rain forests of Madagascar, arrived there possibly more that 25 Ma after the formation of the Malagasy rain forest biome (Figs. 1 and 2). This is at odds with the expectation that the diverse and dominant components of Madagascar’s rain forests should be either early colonists from Africa or pre-existing lineages that adapted to changing conditions in situ during the formation of the biome [6, 12]. How can the success of such a late-arriving lineage be explained?

We speculate that *Canarium*’s success could be due, in part, to the fact that Malagasy forests are unusually stochastic in terms of intra- or inter-annual precipitation and the frequency of tropical cyclones [5]. This climatic variability causes unpredictable patterns in floral phenology and has been hypothesized to play a significant role in shaping the evolutionary trajectory of faunal diversity on the island, including unusual life-history patterns, gigantism, and the paucity of mammalian clades [5]. In contrast, little attention has been paid to the impact of a hyper-variable climate on the evolution of plant communities on the island. It is possible that climatic stochasticity has resulted in lineage turnover in Madagascar’s forests, and that a periodic resetting of the ecological stage has provided the opportunity for late (post-rain forest formation) arrivers to become established and radiate [12, 69]. Such a scenario has been invoked to explain adaptive radiations in other areas with unstable climatic regimes [70], and it would help to reconcile our findings with diversification patterns expected from evolutionary radiation into newly forming biomes [6, 12, 69]. If Malagasy plant communities have, in fact, experienced periodic high rates of lineage turnover, this could also help to explain aspects of the evolution of the associated animals, such as the limited number of frugivorous mammals [5].

## Conclusions

Our findings help explain the origin of a previously undocumented radiation of rain forest trees in Madagascar. More broadly, our study highlights that, contrary to expectations under adaptive radiation theory [12], Malagasy *Canarium* did not arrive from Africa during the formation of the rain forest biome, but rather from the Indo-Australian Archipelago some 20 Ma later. This finding is consistent with the observation that Malagasy rain forests experience hyper-variable climates. Under these circumstances, high rates of local extinction might provide an opportunity for late-arriving lineages to successfully colonize and radiate. The arrival of *Canarium* in Madagascar from the IAA highlights the potential significance of long distance dispersal in establishing pantropical distributions since the Oligocene. Moreover, we suggest that the IAA may have served as a source area for multiple lineages now widespread in tropical forests, a topic deserving further investigation.

### Availability of supporting data

The molecular data supporting the results of this article are available in Genbank (http://www.ncbi.nlm.nih.gov/genbank/), with NCBI gi numbers detailed in Additional file 1: A2 and A3. The phylogenetic data sets are available in the Zenodo repository (https://zenodo.org/, *DOI*10.5281/zenodo.31503).

## Additional file

Additional file 1:
**A1. A2.** voucher & GenBank information. **A3.** GenBank information. **A4.** biogeographic models. **A5.** (**A**) Fossil node dated phylogeny, with Canarieae Fossil. **A5.** (**B**). Fossil node dated phylogeny, without Canarieae Fossil. **A6.** RaxML phylogeny. **A7.** Statistical Dispersal Vicariance Extinction (DEC). **A8.** Biogeobears biogeographic reconstructions. (DOCX 10138 kb)
