# Supplementary material for: The biogeographic origin of a radiation of trees in Madagascar: implications for the assembly of a tropical forest biome
Source: BMC Evol Biol. 2015 Oct 5;15:216. doi: 10.1186/s12862-015-0483-1 (PMC4594639; doi:10.1186/s12862-015-0483-1)
Supplement: Additional file 1: — A1. A2. voucher & GenBank information. A3. GenBank information. A4. biogeographic models. A5. (A) Fossil node dated phylogeny, with Canarieae Fossil. A5. (B). Fossil node dated phylogeny, without Canarieae Fossil. A6. RaxML phylogeny. A7. Statistical Dispersal Vicariance Extinction (DEC). A8. Biogeobears biogeographic reconstructions. (DOCX 10138 kb) [file 12862_2015_483_MOESM1_ESM.docx]

**Additional file, A1**

**Table 1:** Currently recognized genera of Canarieae with the number of species and their geographic distributions indicated. The number of species collected for use in phylogenetic inference and their geographic provenance are also shown. AF = Africa, IAA = Malesia/Asia/Australia, MA = Madagascar, NE = Neotropics.

**Additional file, A2: voucher & GenBank information**

**Table 1.** List of taxa collected by the authors and examined in this study, collection numbers, herbaria where collections are deposited (NY: New York Botanical Garden; MO: Missouri Botanical Garden), and GenBank accession numbers.

**Additional file, A3: GenBank information**

**Table 1.** List of taxa obtained from GenBank for use in the molecular phylogeny with NCBI gi numbers.

**Additional file, A4: biogeographic models**

**Ancestral Area time slice justification:**

These time slices broadly encompass major paleo-ecological and climatic regimes that most likely influenced the biogeographic history of *Canarium* in Madagascar.

*Paleocene-Eocene (61.7-33.9 Ma):*

During this geologic era, much of the earth was warm and moist, facilitating cosmopolitan distributions for many clades confined to the tropics today (Morely 2003). Marine currents favored dispersal from Africa to Madagascar, and much of SE Asia, including the Indo-Australian Archipelago (IAA) had not yet formed (von der Heydt & Dijkstra 2006; Ali & Huber 2010). Additionally, India had not yet collided with mainland Asia and remained relatively close to Madagascar, also facilitating dispersal (Buerki et al. 2013). Madagascar transitions from a moist temperate climate to an arid one (Wells 2003).

*Oligocene- early Miocene (33.9-16 Ma):*

During this geologic era, the boreotropics began contracting, and tropical clades either had to move towards warmer latitudes or adapt to temperate climates (Morely 2003). India drifted further from Madagascar, dispersal from Africa was still possible but becoming less likely via marine pathways, the IAA and associated westward moving warm surface currents began forming (von der Heydt & Dijkstra 2006; Ali & Huber 2010; Buerki et al. 2013). Additionally, this geologic era marks the advent of Madagascar’s moist forest biome (Wells 2003).

*Mid-Miocene-Present (16 Ma – Present):*

During this geologic era, the eastern Tethys seaway closed drastically altering marine currents between Africa and Eurasia (Kennett 1985), the IAA and associated fast moving westward surface currents had formed (running almost directly between the IAA and Madagascar), monsoon pathways had formed (figure 3; Dewar & Richard 2007), India had collided with Eurasia, and marine currents between Africa and Madagascar were no longer conducive to dispersal from the mainland (Ali & Huber 2010; Buerki et al. 2013).

| **TERRESTRIAL MODEL** | | | | | | | |
| --- | --- | --- | --- | --- | --- | --- | --- |
| **16-present** | |  |  |  |  |  |  |
|  | **MA** | **IAA** | **LA** | **IN** | **SP** | **AF** | **NE** |
| **MA** | 1 | 0 | 0 | 0.05 | 0 | 1 | 0 |
| **IAA** | 0 | 1 | 1 | 1 | 1 | 0 | 0 |
| **LA** | 0 | 1 | 1 | 1 | 0 | 1 | 1 |
| **IN** | 0.05 | 1 | 1 | 1 | 0 | 1 | 0 |
| **SP** | 0 | 1 | 0 | 0 | 1 | 0 | 0 |
| **AF** | 1 | 0 | 1 | 1 | 0 | 1 | 0 |
| **NE** | 0 | 0 | 1 | 0 | 0 | 0 | 1 |
| **33.9-16** | |  |  |  |  |  |  |
|  | **MA** | **IAA** | **LA** | **IN** | **SP** | **AF** | **NE** |
| **MA** | 1 | 0 | 0 | 1 | 0 | 1 | 0 |
| **IAA** | 0 | 1 | 1 | 1 | 1 | 0 | 0 |
| **LA** | 0 | 1 | 1 | 1 | 0 | 1 | 0 |
| **IN** | 1 | 1 | 1 | 1 | 0 | 1 | 0 |
| **SP** | 0 | 1 | 0 | 0 | 1 | 0 | 0 |
| **AF** | 1 | 0 | 1 | 1 | 0 | 1 | 0 |
| **NE** | 0 | 0 | 0 | 0 | 0 | 0 | 1 |
| **61.7-33.9** | |  |  |  |  |  |  |
|  | **MA** | **IAA** | **LA** | **IN** | **SP** | **AF** | **NE** |
| **MA** | 1 | 0 | 0 | 1 | 0 | 1 | 0 |
| **IAA** | 0 | 1 | 1 | 1 | 0 | 0 | 0 |
| **LA** | 0 | 1 | 1 | 1 | 0 | 1 | 0 |
| **IN** | 1 | 1 | 1 | 1 | 0 | 1 | 0 |
| **SP** | 0 | 0 | 0 | 0 | 1 | 0 | 0 |
| **AF** | 1 | 0 | 1 | 1 | 0 | 1 | 0 |
| **NE** | 0 | 0 | 0 | 0 | 0 | 0 | 1 |

**Table 1:** Terrestrial model for seven biogeographic areas: Neotropics (NE); Africa (AF); Sundaland and Indochina (IAA); India (IN); Laurasia (LA); Madagascar (MA); and South Pacific (including New Caledonia, Australia, and Papua New Guinea [SP]). The model is divided into three time slices – (1) 56-33.9 Ma; (2) 33.9-16 Ma; and (3) 16 Ma – present. Dispersal rates between biogeographic areas are represented by: 0 (no dispersal); 0.05 (low dispersal probability among areas); and 1 (high dispersal probability among areas).

| **MARINE+TERRESTRIAL MODEL** | | | | | | | |
| --- | --- | --- | --- | --- | --- | --- | --- |
| **16-present** | |  |  |  |  |  |  |
|  | **MA** | **IAA** | **LA** | **IN** | **SP** | **AF** | **NE** |
| **MA** | 1 | 0.05 | 0 | 0.05 | 0.05 | 1 | 0 |
| **IAA** | 1 | 1 | 1 | 1 | 1 | 1 | 0.05 |
| **LA** | 0 | 1 | 1 | 1 | 0 | 1 | 1 |
| **IN** | 1 | 1 | 1 | 1 | 0.05 | 1 | 0 |
| **SP** | 1 | 1 | 0 | 1 | 1 | 1 | 0.05 |
| **AF** | 1 | 0.05 | 1 | 1 | 0.05 | 1 | 1 |
| **NE** | 0 | 0.05 | 1 | 0 | 0.05 | 1 | 1 |
| **33.9-16** | |  |  |  |  |  |  |
|  | **MA** | **IAA** | **LA** | **IN** | **SP** | **AF** | **NE** |
| **MA** | 1 | 1 | 0 | 1 | 1 | 1 | 0 |
| **IAA** | 1 | 1 | 1 | 1 | 1 | 1 | 0.05 |
| **LA** | 0 | 1 | 1 | 1 | 0 | 1 | 0 |
| **IN** | 1 | 1 | 1 | 1 | 0 | 1 | 0 |
| **SP** | 1 | 1 | 0 | 0 | 1 | 0 | 0.05 |
| **AF** | 1 | 1 | 1 | 1 | 0 | 1 | 0.05 |
| **NE** | 0 | 0.05 | 0 | 0 | 0.05 | 0.05 | 1 |
| **61.7-33.9** | |  |  |  |  |  |  |
|  | **MA** | **IAA** | **LA** | **IN** | **SP** | **AF** | **NE** |
| **MA** | 1 | 0 | 1 | 1 | 0 | 1 | 0 |
| **IAA** | 0 | 1 | 1 | 1 | 0 | 0 | 0.05 |
| **LA** | 1 | 1 | 1 | 1 | 0 | 1 | 0 |
| **IN** | 1 | 1 | 1 | 1 | 0 | 1 | 0 |
| **SP** | 0 | 0 | 0 | 0 | 1 | 0 | 0.05 |
| **AF** | 1 | 0 | 1 | 1 | 0 | 1 | 0.05 |
| **NE** | 0 | 0.05 | 0 | 0 | 0.05 | 0.05 | 1 |

**Table 2:** Marine + terrestrial model for seven biogeographic areas: Neotropics (NE); Africa (AF); Sundaland and Indochina (IAA); India (IN); Laurasia (LA); Madagascar (MA); and South Pacific (including New Caledonia, Australia, and Papua New Guinea [SP]). The model is divided into three time slices – (1) 56-33.9 Ma; (2) 33.9-16 Ma; and (3) 16 Ma – present. Dispersal rates between biogeographic areas are represented by: 0 (no dispersal); 0.05 (low dispersal probability among areas); and 1 (high dispersal probability among areas)

**Additional file, A5 (A) Fossil node dated phylogeny, with Canarieae Fossil**


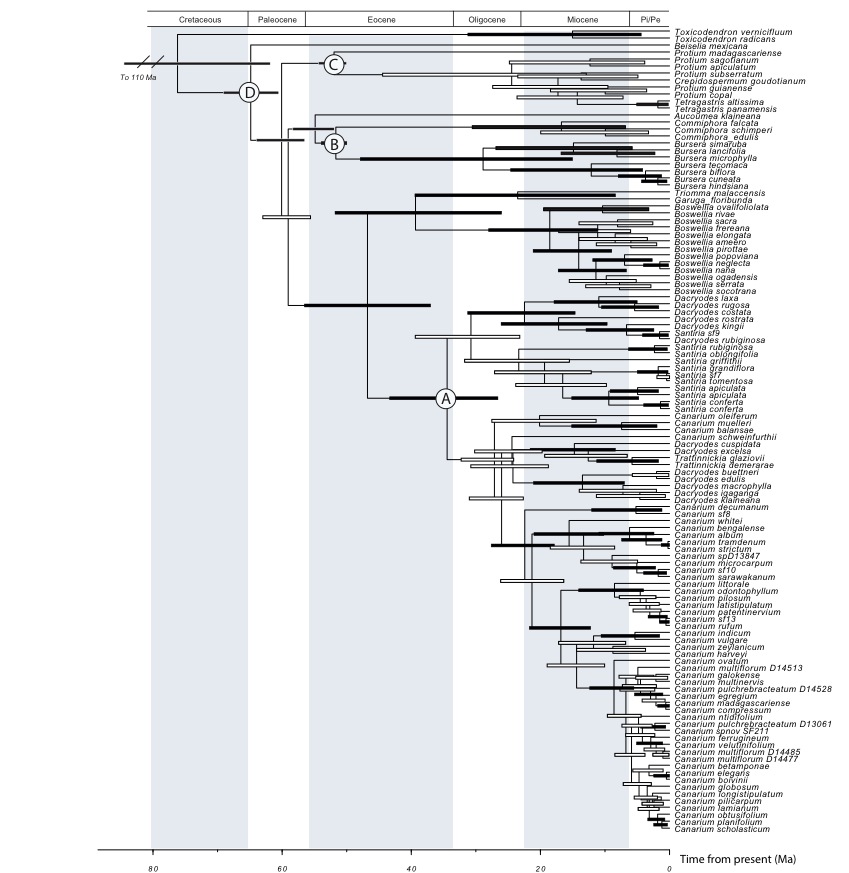


**Figure 1.** Molecular phylogeny of the Canarieae. Maximum clade credibility tree summarizing the results of Bayesian dating analysis in BEAST (fossil calibration nodes marked with letters A-D), with a normal probability prior on node A and lognormal probability priors on nodes B-D, following Fine *et al.* (2014). Bars represent both highest posterior density intervals of the dating analysis as well as Bayesian posterior probabilities (BPP), where black bars represent a BPP of .95 and higher while uncolored bars have a BPP of .94 and below.

**A5 (B) Fossil node dated phylogeny, without Canarieae Fossil**


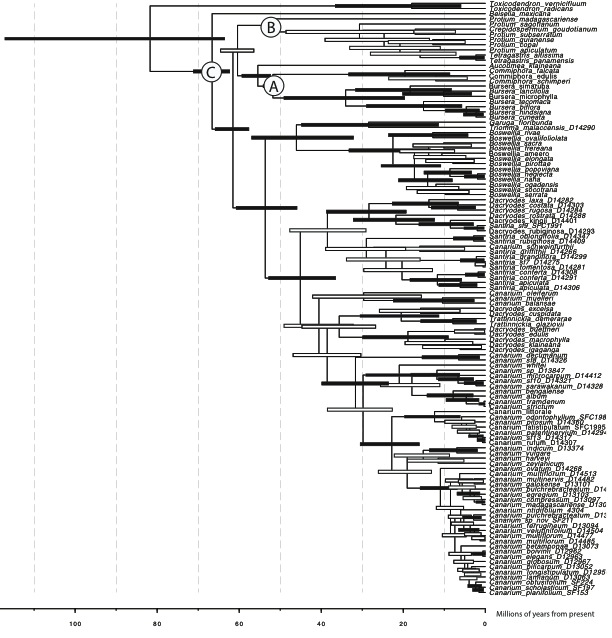


**Figure 1.** Molecular phylogeny of the Canarieae. Maximum clade credibility tree summarizing the results of Bayesian dating analysis in BEAST (fossil calibration nodes marked with letters A-C), with a normal probability prior on node A and lognormal probability priors on nodes B-C, following Fine *et al.* (2014). Bars represent both highest posterior density intervals of the dating analysis as well as Bayesian posterior probabilities (BPP), where black bars represent a BPP of .95 and higher while uncolored bars have a BPP of .94 and below.

**Additional file, A6:** **RaxML phylogeny**

**Figure 1.** Maximum likelihood Canarieae phylogeny constructed in RaxML with 1000 bootstraps. Bootstrap support values are detailed at each node

**Additional file, A7: Statistical Dispersal Vicariance Extinction (DEC)**

**Figure 1.** Statistical dispersal extinction cladogenesis (S-DEC) ancestral area reconstructions using 1000 trees from the posterior distribution of the fossil-tip dated phylogeny. Ancestral areas are color-coded with the legend in the lower right of the figure. Areas are coded as: Neotropics (NE); Africa (AF); Sundaland and Indochina (IAA); India (IN); Laurasia (LA); Madagascar (MA); and South Pacific (including New Caledonia, Australia, and Papua New Guinea [SP]).

**Additional file, A8: Biogeobears biogeographic reconstructions**

| Model^*^ | LnL^1^ | P^2^ | d^3^ | e^4^ | j^5^ | AIC^6^ | dAIC^7^ | W^8^ |
| --- | --- | --- | --- | --- | --- | --- | --- | --- |
| SD (BAY) | -134.699 | 2.000 | 0.005 | 0.039 | 0.000 | 273.399 | 55.515 | 8.81E-13 |
| SD + j (BAY) | -133.080 | 3.000 | 0.005 | 0.035 | 0.005 | 272.159 | 54.276 | 1.64E-12 |
| LDD (BAY) | -127.897 | 2.000 | 0.004 | 0.028 | 0.000 | 259.794 | 41.910 | 7.93E-10 |
| LDD + j (BAY) | -121.394 | 3.000 | 0.003 | 0.017 | 0.009 | 248.787 | 30.904 | 1.95E-07 |
| LDD + j (DEC) | -116.717 | 3.000 | 0.007 | 0.003 | 0.000 | 239.434 | 21.550 | 2.09E-05 |
| SD + j (DEC) | -114.831 | 3.000 | 0.011 | 0.004 | 0.000 | 235.661 | 17.778 | 1.38E-04 |
| SD (DEC) | -114.830 | 2.000 | 0.011 | 0.004 | 0.000 | 233.659 | 15.775 | 3.75E-04 |
| LDD (DEC) | **-106.942** | **2.000** | **0.006** | **0.002** | **0.000** | **217.884** | **0.000** | **0.999** |

**Table 1**. Biogeographic model fits for the fossil-node calibrated tree

^*^ SD refers to models incorporating terrestrial and short distance marine pathways of dispersal, while LDD also incorporates long distance marine dispersal pathways; the addition of a + j, indicates that colonization pathways were inferred with the founder event parameter. BAY refers to models estimated using a likelihood version of BayArea while DEC refers to models estimated using dispersal extinction cladogenesis.

^1^ Log likelihood

^2^ Number of parameters in the model

^3^ Estimated dispersal rate

^4^ Estimated extinction rate

^5^ Estimated founder event speciation rate

^6^ AIC score

^7^ difference in AIC between the best fit and other candidate models

^8^ AIC weight, or the relative likelihood of the model

Results of colonization pathway estimations for SD and LDD models with inferences using DEC versus DEC + j, and BayArea vesus BayArea + j. The bolded line corresponds to best fitting model, which represent LDD events estimated under DEC, and account for .999 of the Akaike weights. Results of colonization pathway estimations for the fossil-tip dated phylogeny can be found in Table 1 of the main text.

**Additional file A8 (A-H): reconstructions using the fossil-tip phylogeny (ordered according to AIC scores, as in Table 1 of the main text)**

**A8, (A)**

**A8, (B)**

**A8, (C)**

**A8, (D)**

**A7, (E)**

**A7, (F)**

**A8, (G)**

**A8, (H)**

**Additional file A8 (I-P): reconstructions using the fossil-node phylogeny (ordered according to AIC scores, as in Table 1 of Additional file A8)**

**A8, (I)**

**A8, (J)**

**A8, (K)**

**A8, (L)**

**A8, (M)**

**A8, (N)**

**A8, (O)**

**A8, (P)**

**Figure 1.** Ancestral area reconstructions in biogeobears (Matzke 2014) using both the fossil tip dated phylogeny (A8, (A-H)) and the fossil-node dated phylogeny (A8, (I-P)). All ancestral areas are ordered according to their AIC weights as listed in Table 1 of the main text and Table 1 of Additional File, A8. Ancestral areas were inferred on each tree using a variety of inference methodologies such as dispersal extinction cladogenesis (DEC) (Ree et al. 2005; Ree & Smith 2008), DEC + founder event speciation (j) (Matzke 2014), BayArea (Landis 2013) and BayArea + j (Matzke 2014). Each inference method is noted at the top of each reconstruction (A-P). Additionally, inferences with each reconstruction methodology were done twice: once for the Marine + Terrestrial model (in the text, this is referred to as the LDD + terrestrial model) and once for the Terrestrial model (in the text, this is referred to as the SD + terrestrial model), the model used is also noted at the top of each phylogeny, along with the maximum number of areas (7), Log Likelihood of the model (LnL), and rates dispersal (d), extinction (e), and j (when applicable) (A-P). The seven biogeographic areas are colored and coded at each phylogeny’s tips and in combinations at nodes as follows: **(1)** Africa (AF) colored in red; **(2)** Sundaland and Indochina (SI) colored in teal; **(3)** Neotropics (N) colored in pink; **(4)** India (I) colored in yellow; **(5)** Laurasia (LA) colored in green; **(6)** Madagascar (M) colored in blue; **(7)** South Pacific (including New Caledonia, Australia, and Papua New Guinea [NC]) colored in orange.

**References:**

Ali, J.R. & Huber, M. 2010 Mammalian biodiversity on Madagascar controlled by ocean currents. *Nature* **463**, 653-656.

Buerki, S., Devey, D.S., Callmander, M.W., Phillipson, P.B. & Forest, F. 2013 Spatio‐temporal history of the endemic genera of Madagascar. *Botanical Journal of the Linnean Society* **171**, 304-329.

Dewar, R.E. & Richard, A.F. 2007 Evolution in the hypervariable environment of Madagascar. *Proceedings of the National Academy of Sciences of the United States of America* **104**, 13723-13727. (doi:10.1073/pnas.0704346104).

Drummond, A.J., Suchard, M.A., Xie, D. & Rambaut, A. 2012 Bayesian phylogenetics with BEAUti and the BEAST 1.7. *Molecular biology and evolution* **29**, 1969-1973.

Kennett, J.P. 1985 *The Miocene ocean: paleoceanography and biogeography*, Geological Society of America.

Landis, M.J., Matzke, N.J., Moore, B.R. & Huelsenbeck, J.P. 2013 Bayesian analysis of biogeography when the number of areas is large. *Systematic biology*, syt040.

Matzke, N.J. 2014 Model selection in historical biogeography reveals that founder-event speciation is a crucial process in island clades. *Systematic biology*, syu056.

Morley, R.J. 2003 Interplate dispersal paths for megathermal angiosperms. *Perspectives in Plant Ecology, Evolution and Systematics* **6**, 5-20. (doi:10.1078/1433-8319-00039).

Ree, R.H., Moore, B.R., Webb, C.O. & Donoghue, M.J. 2005 A likelihood framework for inferring the evolution of geographic range on phylogenetic trees. *Evolution; international journal of organic evolution* **59**, 2299-2311.

Ree, R.H. & Smith, S.A. 2008 Maximum likelihood inference of geographic range evolution by dispersal, local extinction, and cladogenesis. *Systematic Biology* **57**, 4-14.

Stamatakis, A. 2006 RAxML-VI-HPC: maximum likelihood-based phylogenetic analyses with thousands of taxa and mixed models. *Bioinformatics* **22**, 2688-2690.

von der Heydt, A. & Dijkstra, H.A. 2006 Effect of ocean gateways on the global ocean circulation in the late Oligocene and early Miocene. *Paleoceanography* **21**.

Wells, N. 2003 Some hypotheses on the Mesozoic and Cenozoic paleoenvironmental history of Madagascar. *The natural history of Madagascar*, 16-34.
